# Root-Lesion Nematodes Suppress Cabbage Aphid Population Development by Reducing Aphid Daily Reproduction

**DOI:** 10.3389/fpls.2016.00111

**Published:** 2016-02-10

**Authors:** W. H. G. Hol, Ciska E. Raaijmakers, Ilse Mons, Katrin M. Meyer, Nicole M. van Dam

**Affiliations:** ^1^Terrestrial Ecology, Netherlands Institute of Ecology (NIOO-KNAW)Wageningen, Netherlands; ^2^Department of Ecosystem Modelling, University of GöttingenGöttingen, Germany; ^3^German Centre for Integrative Biodiversity Research (iDiv) Halle-Jena-LeipzigLeipzig, Germany; ^4^Institute of Ecology, Friedrich Schiller University JenaJena Germany; ^5^Molecular Interaction Ecology, Institute of Water and Wetland Research, Radboud UniversityNijmegen Netherlands

**Keywords:** aboveground-belowground interactions, induced responses, individual-based population model, *Brassica*, root-lesion nematode, root-knot nematode, cabbage aphid

## Abstract

Empirical studies have shown that belowground feeding herbivores can affect the performance of aboveground herbivores in different ways. Often the critical life-history parameters underlying the observed performance effects remain unexplored. In order to better understand the cause for the observed effects on aboveground herbivores, these ecological mechanisms must be better understood. In this study we combined empirical experiments with a modeling approach to analyze the effect of two root feeding endoparasitic nematodes with different feeding strategies on the population growth of the aboveground feeding specialist aphid *Brevicoryne brassicae* on *Brassica nigra*. The aim was to test whether emerging differences in life history characteristics (days until reproduction, daily reproduction) would be sufficient to explain observed differences in aphid population development on plants with and without two species of nematodes. Aphid numbers were lower on plants with *Pratylenchus penetrans* in comparison to aphid numbers on plants with *Meloidogyne* spp. A dedicated experiment showed that aphid daily reproduction was lower on plants with *P. penetrans* (3.08 offspring female^–1^ day^–1^) in comparison to both uninfested plants and plants with *Meloidogyne* spp. (3.50 offspring female^–1^ day^–1^). The species-specific reduction of aphid reproduction appeared independent of changes in amino acids, soluble sugars or the glucosinolate sinigrin in the phloem. An individual-based model revealed that relatively small differences in reproduction rate per female were sufficient to yield a similar difference in aphid populations as was found in the empirical experiments.

## Introduction

Plants are the primary food source on earth for a wide range of aboveground and belowground organisms. To defend themselves against this wide range of herbivores and pathogens, plants possess a large arsenal of defense strategies (Meldau et al., 2012). Many plants increase their level of defense upon damage by an herbivore. Induced defense responses have been found in over 100 plant species. Compared to constitutive defenses, they have the advantage that they can be aimed specifically at the attacker, amongst others based on differences in feeding patterns or cues in the saliva (Erb et al., 2012; Maffei et al., 2012). Induced defense responses can be triggered both by aboveground and belowground herbivores. It has been shown that, depending on the feeding type and species of herbivore, different hormonal signaling pathways are activated upon damage by herbivores or pathogens. Cross talk between these pathways eventually will determine the nature of the defense response (Pieterse et al., 2012). The signaling hormones involved in activating the defense responses are transported throughout the plant, thereby changing the defense levels in undamaged systemic organs as well (Erb et al., 2009). Consequently, the defense responses triggered by a root herbivore may indeed affect the performance of shoot herbivores, and vice versa (Wurst and van der Putten, 2007; Kaplan et al., 2008; Vandegehuchte et al., 2010; Erb et al., 2011; van Dam and Heil, 2011; Kutyniok and Müller, 2012). Because of the specificity of the induced response to different species of herbivores, the direction of the interaction between aboveground and belowground herbivores strongly depends on the feeding habit of the herbivores involved (van Dam and Heil, 2011). Root chewing herbivores, for example, generally have a negative effect on their aboveground counterparts whereas the effects of root herbivores on sap-sucking shoot herbivores, such as aphids may be more diverse (Kaplan et al., 2008; Johnson et al., 2012).

In many ways, root feeding nematodes and shoot feeding aphids are analogs: both are serious plant pests with a sucking-piercing feeding habit. They also intimately interact with their host plant by injecting saliva into the plant. The saliva contains various compounds and proteins, such as cellulases and pectinases, which serve to facilitate penetration of the aphid’s stylet or movement of the nematode itself through plant tissues (Jones et al., 2013; Will et al., 2013). Moreover, other salivary constituents are needed to create a local sink for plant resources at the feeding site (Gheysen and Mitchum, 2011; Will et al., 2013). On the other hand, there are also distinct differences: many plant-parasitic nematodes are endoparasitic and remain inside the root tissue for a large part of their life cycle (Jones et al., 2013). During the endoparasitic phase, root knot nematodes (e.g., *Meloidogyne*) and cyst nematodes (*Heterodera*) are sedentary, and establish specific feeding structures requiring remodeling of plant root tissues. Root-lesion nematodes (e.g., *Pratylenchus*) remain mobile and feed on individual root cortex cells (Jones et al., 2013). This results in very different damage dynamics which may lead to distinct plant responses (Wondafrash et al., 2013). Moreover, the salivary compounds injected by the different nematode species may also be used by the plant to recognize the species that is feeding and to respond accordingly; salivary components of *Myzus persicae* induced defense responses in *Arabidopsis thaliana* (de Vos and Jander, 2009). Similar to aboveground herbivory (Bidart-Bouzat and Kliebenstein, 2011), different feeding styles of belowground herbivores may result in the triggering of different signaling pathways, which may also have consequences for the effect on aboveground herbivores feeding on the same plant (Wondafrash et al., 2013). For nematode-aphid interactions, it was found by Kaplan et al. (2011) that the ability of the generalist aphid *M. persicae* to create a nutrient sink was not affected by *Meloidogyne incognita* infestation (Kaplan et al., 2011). However, this has not been assessed for other nematode species, such as *Pratylenchus penetrans*, or the specialist aphid *Brevicoryne brassicae*.

Even without knowing the exact molecular or chemical mechanisms, it is clear that aboveground-belowground interactions can have serious ecological consequences for all parties involved. Field studies have shown that the presence of small belowground herbivores, such as plant pathogenic nematodes, may have significant effects on large-scale processes such as plant succession (De Deyn et al., 2003). Manipulative studies in greenhouses or mesocosms also demonstrate that the presence of specific root herbivores may have significant effects on the performance of chewing or sucking aboveground herbivores (van Dam et al., 2005; Wurst and van der Putten, 2007; Kaplan et al., 2008; Hol et al., 2010; Erb et al., 2011; Johnson et al., 2012). For chewing herbivores, the performance is mostly assessed in terms of weight gain or survival, whereas for aphids population growth is the most relevant performance parameter (Bezemer et al., 2005; Wurst and van der Putten, 2007; Hol et al., 2013). The advantage of using population growth as a measure is that it is relatively natural: the herbivores can choose where to feed on the plant and it will include density-dependent effects, such as the emergence of winged adults at high aphid densities. However, it is hard to obtain reliable counts of aphids over a substantial period of time on sufficient numbers of replicates, as the populations of these insects may grow very rapidly and can produce hundreds of individuals per plant within a period of a few weeks (Hughes, 1963). A complementary approach to gain a more detailed insight in aboveground-belowground interactions would be to look at individuals, e.g., confined to clip cages, to obtain precise estimates of life-history parameters such as pre-reproductive period, longevity, and reproductive rates (e.g., Ellis et al., 1996; Stafford et al., 2012; Tariq et al., 2013). To scale these more precise, yet local and often short-term, estimates of life-history parameters up to effects on the population level, the data can be used to parameterize models that simulate population growth over time. Comparisons of simulated and actual population growth can indicate whether effects of belowground organisms can be fully explained by changes in individual life history parameters of aboveground herbivores or whether alternative explanations apply. Similar model simulations based on parameters obtained from greenhouse experiments have been used successfully to test highly complex interactions between above- and belowground organisms associated with plants (Meyer et al., 2009a). Using models to analyze such interactions has the advantage that sample size and statistical power can be increased to levels that are hard to attain in experimental set-ups (Meyer et al., 2009b).

In this study, we apply a combined experimental and modeling approach to analyze the effect of two types of root-feeding endoparasitic nematodes on the population growth of the specialist aboveground feeding aphid *B. brassicae* on the plant species *Brassica nigra*. A field survey showed that *B. nigra* plants in the Netherlands are commonly colonized by a range of plant parasitic nematodes with *Pratylenchus* spp. occurring frequently and *Meloidogyne* spp. only incidentally (**Table 1**). *B. brassicae* aphids are also commonly found on *B. nigra* (Le Guigo et al., 2012; Bischoff and Hurault, 2013). First we experimentally assessed the effects of two nematode species on aphid population growth as well as on some critical life-history parameters of aphids. Then, using a modeling approach, we validated this effect by simulating the effects on aphid population growth, thereby including published data on additional life history parameters of *B. brassicae*. An individual-based model was developed to simulate aphid population growth on *B. nigra* under controlled greenhouse conditions, in the absence of natural enemies such as predators or parasitoids, but including stochastic effects for mortality and reproductive rate of the aphid. The aim was to test whether the life history characteristics (daily probability of reproduction, average number of offspring per day) as determined in the life history experiment would be sufficient to explain the observed differences in aphid population numbers on plants with and without nematodes in the greenhouse experiment. Finally, we analyzed the chemical composition, i.e., amino acids, sugars and sinigrin content, of the phloem of *B. nigra* to gain insight in the nutritional mechanisms behind the observed effect. Sinigrin is the main glucosinolate in *B. nigra* which is known to serve as an inducible defense against a wide range of generalist herbivores (Lankau, 2007). It is known to be excreted in the nectar and transported in the phloem (Bruinsma et al., 2014).

**Table 1:** Average numbers of all nematodes, the relative percentage of plant-parasitic nematodes, and numbers of specific groups of plant-parasitic nematodes found in roots and rhizosphere soil samples of *Brassica nigra*.

	Elderveld	Heteren	Zetten
	51°57′N,5°52′E	51°57′N,5°45′E	51°55′N,5°43′E
Total number of nematodes per plant sample	348	848	563
	(68–1250)	(80–1485)	(121-1859)
Percentage plant-parasitic nematodes of total	6.2%	72.8	47.2 %
	(0–17.6)	(46.6–90.0)	(18.2-70.4)
*Paratylenchus*	n.d.	51	229
		(2–132)	(11-891)
Ectoparasites rest	20	22	4
	(0–121)	(0–77)	(0-11)
*Pratylenchus* spp.	6	370	108
	(0–22)	(23–1023)	(0-407)
Tylenchidae	17	116	7
	(0–99)	(29–319)	(0-11)
*Meloidogyne* spp.	n.d.	20	n.d.
		(0–55)	
*Heterodera*	n.d.	2	n.d.
		(0–11)	

*The plants were collected in three Dutch populations (column headings give names plus geolocation; n = 5 plants per population, except Elderveld n = 6) in the summer of 2004. Numbers between brackets give the range (minimum – maximum) for each count. n.d., not detected.*

## Materials and Methods

### Naturally Occurring Nematode Communities on *B. nigra*

In three naturally occurring roadside populations nearby Heteren, The Netherlands, five or six randomly chosen *B. nigra* root systems were dug out as completely as possible. The roots were severed from the shoots using clippers, and the remaining bulk soil was removed by gently shaking the roots before bagging them. After transport to the lab, the roots were cleaned from remaining soil with tap water and cut into small pieces (2–4 cm). The root pieces were placed in a mist chamber (van Bezooijen, 2006) and extracted for nematodes for 96 h. The samples were stored at 4°C until identification of the different species under a reverse light binocular microscope [50–200 × magnification (Brinkman et al., 2004)].

### Nematode and Insect Cultures

Ten to twenty *B. brassicae* individuals were collected in the common garden of the Centre for Terrestrial Ecology, NIOO-KNAW (Heteren, The Netherlands) and placed on *B. nigra* plants in the greenhouse. The populations were maintained for at least two generations before they were used for experiments.

A starting culture of *P. penetrans* was originally obtained from HZPC Holland BV (Joure, the Netherlands) and maintained on 10 L containers with rye (*Avena sativa*) plants grown on plain sand and Hoagland solution. *Meloidogyne hapla* was obtained from the same source and maintained on tomato plants (c.v. Moneymaker). *M. incognita* was originally obtained from the laboratory of Nematology (Wageningen, The Netherlands) and maintained on tomato plants (c.v. Moneymaker). Since the two species of *Meloidogyne* that were used in the experiments were very similar in their effects on aphids, we will uniformly refer to these treatments as *Meloidogyne* treatments. Before infesting the experimental plants, nematodes were extracted from roots and sand in soil cores drawn from these pots (Brinkman et al., 2004). Two days after extraction, root and soil extracts were pooled and an aliquot was counted using a light microscope to determine nematode density in the extract.

### Empirical Aphid Population Development Experiment – Effects of Nematode Species

*Brassica nigra* seeds, collected from a population of open pollinated plants (>10) grown in an experimental field in Wageningen in 2004, were germinated on glass beads and water in a climate cabinet set at 22°C day/16°C night, 16 h light. After 1 week, the seedlings were planted individually in tall 2.2 L pots (11 cm × 11 cm × 21.5 cm) filled with gamma-radiated (>25 KGray, Isotron, Ede, The Netherlands) sterile sand and covered with aluminum foil – except for a hole in the middle through which the seedling could grow – to reduce water loss due to evaporation. A ∼4 cm long piece of a drinking straw was inserted in the soil next to the seedling to facilitate nematode infestation later on. The pots with seedlings were placed in a glasshouse kept at 21°C D/16°C N under ambient light conditions that were supplied with sodium lamps to maintain the minimum photosynthetically active radiation at 225 μmol.m^–2^.s^–1^ for 16 h per day. The pots were supplemented with 3P Hoagland solution and water as described in van Dam et al. (2005) two or three times a week to maintain 14% soil moisture (w/w) and optimize plant growth. After 3 weeks of growth, the plants were either infested with 400 *P. penetrans* or 400 *M. incognita* individuals per plant (*n* = 22 per treatment group) by pipetting 5 ml of nematode extract in the straw inserted next to the plant. Control plants (*n* = 22) were mock inoculated with 5 ml of tap water. After another 3 weeks, the pots and plants were enclosed individually in hanging spherical nets (diameter 25 cm, height 1.5 m) that were closed at the bottom with a rubber band around the pot. Per plant, five *B. brassicae* individuals of mixed ages were placed with a soft paint brush on a fully expanded leaf via the zippered opening in the side of the hanging net cages. The numbers of aphids were counted at 3, 7, 10, 14 days after infestation with aphids. The numbers of aphids per plant were square root transformed before analysis to meet assumptions of normality and homogeneity of variances. Two data points in the *Pratylenchus* treatment were discarded as the SPSS outlier analysis indicated they were significant outliers, leaving 20 replicates in this treatment group. The remaining data were analyzed using Repeated Measures ANOVA followed by protected contrast analysis (IBM SPSS Statistics release 20.0.0).

Chemical analysis – The concentrations of amino acids and sugars in the first two young fully expanded leaves and in the phloem 2 and 5 weeks after aphid infestation were measured according to van Dam and Oomen (2008) and Hol et al. (2013). In the phloem, glucosinolate concentrations were measured on pools of 3–4 samples (*n* = 2–3) to obtain samples with detectable levels of sinigrin. The amino acids and sugars in the leaves and phloem after 2 and 5 weeks were analyzed with MANOVA, *n* = 7 for 2 weeks, *n* = 8–11 for 5 weeks. The glucosinolate data from the pooled phloem samples were arcsine square root transformed to obtain normality for the residuals. To investigate to what extent pooling of phloem samples may have affected the result, we performed a theoretical pooling of the data from the amino acids and sugar concentrations in the phloem by calculating the mean concentrations for the same sets of plants whose phloem samples were pooled for the sinigrin analysis.

### Life History Parameters of Aphids on Nematode-Infested Plants

Plants were grown on sterile sand as above, and infested with 400 *P. penetrans* (*n* = 23) or 400 *M. hapla* (*n* = 23) 3 weeks after transfer to the pots. Another 22 plants served as uninfested controls. After another 3 weeks, each plant received two clip cages (5 cm diameter) on two fully expanded leaves. Each clip cage contained one neonate *B. brassicae*. Neonates were obtained by isolating adult aphids in individual Petri dishes 1 day before the experiment started. When cages appeared empty 1 day later, the neonates were considered to be lost or escaped and replaced. This was accounted for in the calculations to assess “first day till reproduction.” Thereafter, no replacements took place. Each day, every single cage was checked for survival and reproduction up till 16 days after the neonates were placed on the plants. After the original neonates had started to reproduce, newly emerged neonates were removed from the cages each day after they had been counted. The numbers of nymphs produced per each reproducing female per day from the second day of reproduction onwards were averaged per treatment. Significant differences between the treatment groups were identified using Wilcoxon rank sum test with continuity correction using R 3.0.0. (R Core Development Team, 2013) followed by correction for multiple comparisons according to Holm (1979).

### Aphid Population Growth Simulation Model

We developed an individual individual-based computer model of aphid population growth. The model was implemented in C# with Microsoft Visual C# 2010 Express and the full code of the model is available as Supplementary Material. The model description follows the ODD (Overview, Design concepts, Details) protocol for individual-based models (Grimm et al., 2006, 2010).

#### Purpose

The model was developed to investigate whether individual life-history parameters of aphids are sufficient to explain patterns of aphid population growth.

#### Entities, state variables, and scales

The entities in the model are aphid individuals. State variables are aphid age and livelihood, as well as aphid population size. Scales include 1 day as time step and 24 days as temporal extent, corresponding to the time frame of the empirical experiment. The model is not spatially explicit.

#### Process overview and scheduling

Initialization of the aphid population is followed by age-dependent mortality, age-dependent reproduction, and aging of aphid individuals, as well as writing output and updating the time step.

#### Design concepts: Basic principles

The model is based on the principle that simulated and real-world experiments can be compared to expand the scope of ecological conclusions from the real-world experiments (Meyer et al., 2009b). *Emergence* – Aphid population dynamics emerges from the model. *Sensing* – Individuals have information on their own age and livelihood status and, indirectly via changed parameter values, about the presence of nematodes. *Stochasticity* – Initial aphid age is randomly drawn from a uniform distribution. Mortality and reproduction include stochasticity by comparing a random number drawn from a uniform distribution with the respective mortality or reproduction probability. Number of offspring is randomly drawn from a Poisson distribution. *Observation* – Aphid population size is collected at the end of each time step and output is produced on total population size at time of harvest (after 14 days). Simulation runs of the model correspond to the replication of an empirical experiment so that average daily population sizes can be obtained for different levels of replication. Averages and standard errors were calculated over 22 simulation runs to assure comparability with the aphid population growth experiment.

#### Initialization

The model is initialized with a population of five alive aphids, each with an age that is drawn at random from a uniform distribution between 10 and (including) 24 days. For the greenhouse experiment adult aphids were selected from a mixed population and thus we assume that the age of the aphids at the start of the experiment may have varied from 10 to 25 days. Aphid longevity of 25 days is also derived from data on European *B. brassicae* aphids in Hughes (1963) where adult lifespan ranged from 18.5 days at 17°C to 12.2 days at 23.8°C. Given the average temperature of 20.9°C during the greenhouse experiment, we estimated adult longevity at 15 days and the duration of one generation at 25 days since most individuals were reproducing at day 10.

#### Input data

The model does not contain any time-dependent input data.

#### Submodels

The mortality submodel is implemented with a simple stochastic procedure comparing a random number from a uniform distribution for each live aphid individual to an age-dependent mortality probability (see **Table 2** and Supplementary Table S1 for reference parameter values). Mortalities were derived from Pavela et al. (2004) and Lashkari et al. (2007) where empirically assessed *B. brassicae* survival declined linearly with age. This translates into a fixed mortality of 4% per day in our model: each individual has a daily probability to die of 4% and any surviving individuals die the latest at day 25. The reproduction submodel includes stochasticity in the same way as the mortality submodel. Reproduction probability is age-dependent and was derived from the life-history experiment (**Tables 2** and **3**, Supplementary Table S1, Supplementary Figure S1). If an individual is reproducing, the number of offspring is drawn from a Poisson distribution with mean 2–3.51 aphids per day, depending on age and also derived from the life-history experiment (**Table 2**; Supplementary Figures S2 and S3). In the empirical experiment, the mean number of offspring per individual per day varied depending on the presence of nematodes on the plant (**Table 3**). We use this range of variation in our simulation experiments (see below) to indirectly include the effect of nematodes on aphids. Based on Ellis et al. (1996), the number of offspring is lower for aphids of 18–21 days and zero for aphids older than 21 days (Supplementary Table S1). The aging submodel increments the age of all aphid individuals by 1 day per time step.

**Table 2:** Parameters used for the aphid population growth simulation model.

Name	Unit	Range	Reference	Details
Start population	Individuals	5	Greenhouse Exp1	
Start age	days	10–25	Greenhouse Exp1	Drawn at random
Daily mortality	Percentage	4	Pavela et al., 2004	Linear declining survival with age
Reproductive status		Yes-no	Life history Exp2	Age-dependent probability^a^
Reproduction	Individuals	2.75–3.51	Life history Exp2 Ellis et al., 1996	Poisson distribution, age-dependent^b^ Power analysis: 2.75–3.45, in steps of 0.05
Maximum age	Days	25	Hughes, 1963	
Duration	Days	14	Greenhouse Exp1	

^a^*Probability to reproduce is age-dependent, see Supplementary Table S1 in Supplementary Material 1 for parameterization.*

^b^*Daily reproduction is age-dependent and the maximum mean depends on nematode presence, see Supplementary Table S1 in Supplementary Material 1 for parameterization.*

**Table 3:** Effect of nematode infestation on survival and reproduction of *Brevicoryne brassicae* on *Brassica nigra* plants infested with different species of nematodes (*Melodoigyne hapla*, *Pratylenchus penetrans*) or mock infested with water (Control).

Treatment	% Drop	% Dead	Maturation	Reproduction
Control	32	7	10.2 (0.2)	3.50 (0.13)^a^
*M. hapla*	32	4	10.0 (0.1)	3.51 (0.13)^a^
*P. penetrans*	30	4	10.3 (0.2)	3.08 (0.13)^b^

*n = 20–22 per treatment. % drop = % of aphids that dropped during counting; % dead = % aphids recorded dead on the leaf; Maturation = average number of days from neonate till first reproduction (+SE); Reproduction = average number of offspring per surviving aphid per day after the first day of reproduction (+SE). Letters indicate significant differences between groups (Wilcoxon rank sum test with continuity correction).*

#### Simulation experiments

We conducted simulation experiments with 22 and 50 runs each, corresponding to the level of replication of an empirical experiment, for three different scenarios. The scenarios differed in their parameterization to represent the two nematode treatments and the control treatment from the life history experiment (**Table 3**). We compared model and experiment outputs to validate the model and to find out whether individual life history characteristics can explain aphid population dynamics under the influence of nematodes. For 1000 simulation experiments we tested with ANOVA whether or not the difference between control and *Pratylenchus* treatment would be significant. Power was calculated as the number of significantly different outcomes/1000. We conducted this power analysis for 22 and 50 replicates to demonstrate the required replication to obtain significant differences in a real-world experiment, all else being equal to the experimental setup of this study. Data available from the Dryad Digital Repository (Hol et al., 2016).

## Results

**FIGURE 1:**
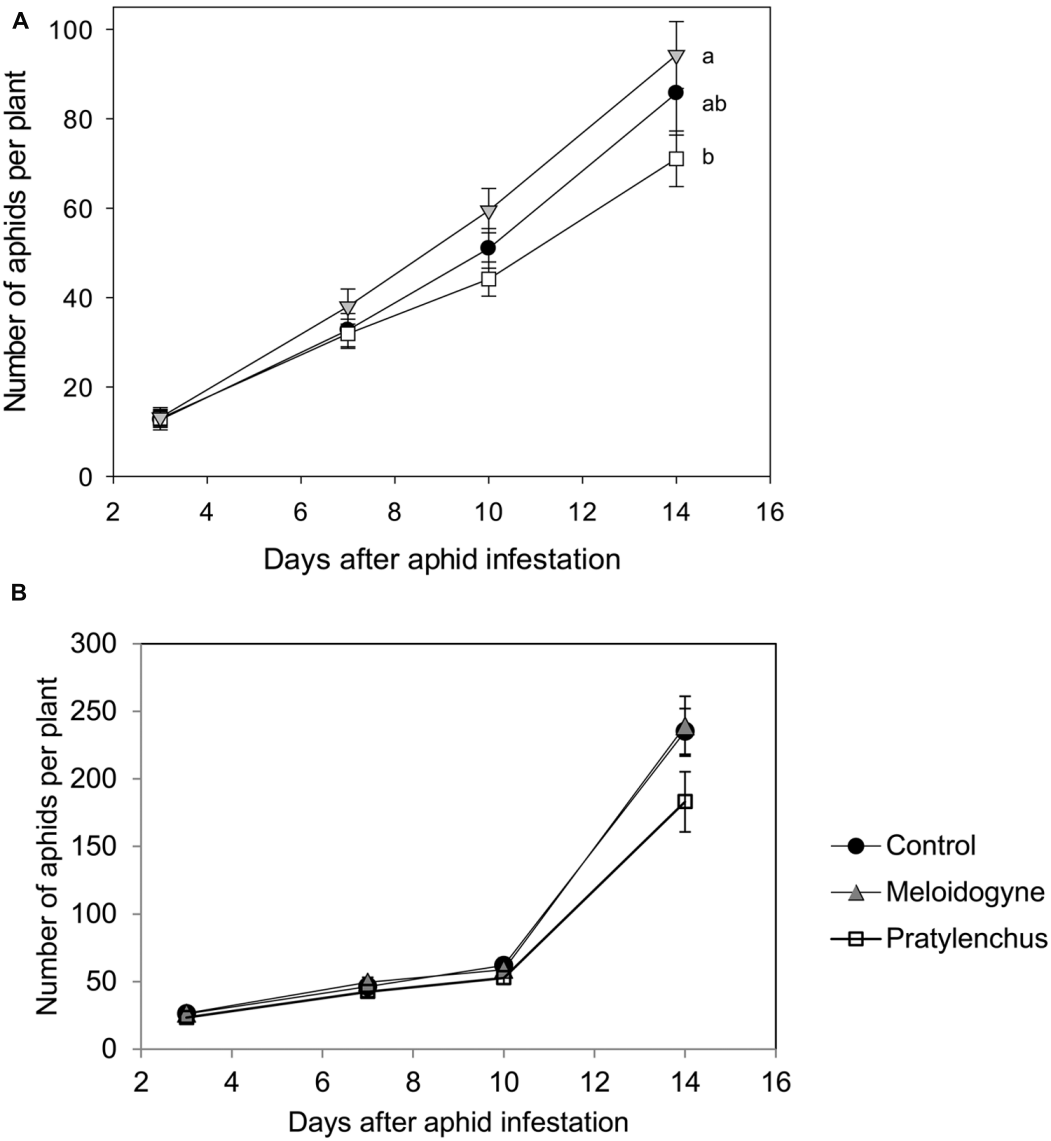
**Average numbers of aphids (+SE) on plants infested with *Pratylenchus penetrans* (white squares), *Meloidogyne incognita* (gray triangles) or mock infested with water (black circles). (A)** Aphid population development in the greenhouse experiment. *n* = 20–22 Different letters indicate significant differences in aphid population development (Repeated measures ANOVA followed by contrast analysis). **(B)** Aphid numbers in a simulation experiment, parameterized to resemble the greenhouse experiment in 1A.

When aphids grew on nematode-infested plants in the greenhouse, their numbers per plant after 14 days of infestation varied depending on nematode species (**Figure 1A**). Aphid population development differ significantly between the different treatment groups (Repeated measures ANOVA time^∗^treatment effect *F*_2,61_ = 3.689, *P* = 0.031). Eventually, the numbers of aphids at the end of the experiment were significantly lower on plants with *P. penetrans* than on plants infested with *M. incognita* (**Figure 1A**). The life-history experiment showed that the maturation time, i.e., the number of days until first reproduction of *B. brassicae* was not significantly different between treatments (**Table 3**). Most females produced their first offspring between day 8 and 10 and continued to reproduce until day 16, when the experiment was ended (Supplementary Figure S1). In contrast, the daily reproduction per aphid was significantly lower for neonate aphids confined to a plant with *P. penetrans* in comparison to those reared on plants with *M. hapla* or control plants without nematodes [**Table 3**, Wilcoxon rank sum test, Control vs. *Pratylenchus*, P (after Holm’s correction for multiple comparisons (Holm, 1979) = 0.048; *Meloidogyne* vs. *Pratylenchus*, *P* = 0.042; Control vs. *Meloidogyne*, *P* = 0.93]. For aphids reared on control plants or *M. hapla* infested plants, the daily reproduction per aphid was on average around 3.50 nymphs per female per day, whereas on plants infested with *P. penetrans* the females produced on average 3.08 offspring per female per day (**Table 3**).

**FIGURE 2:**
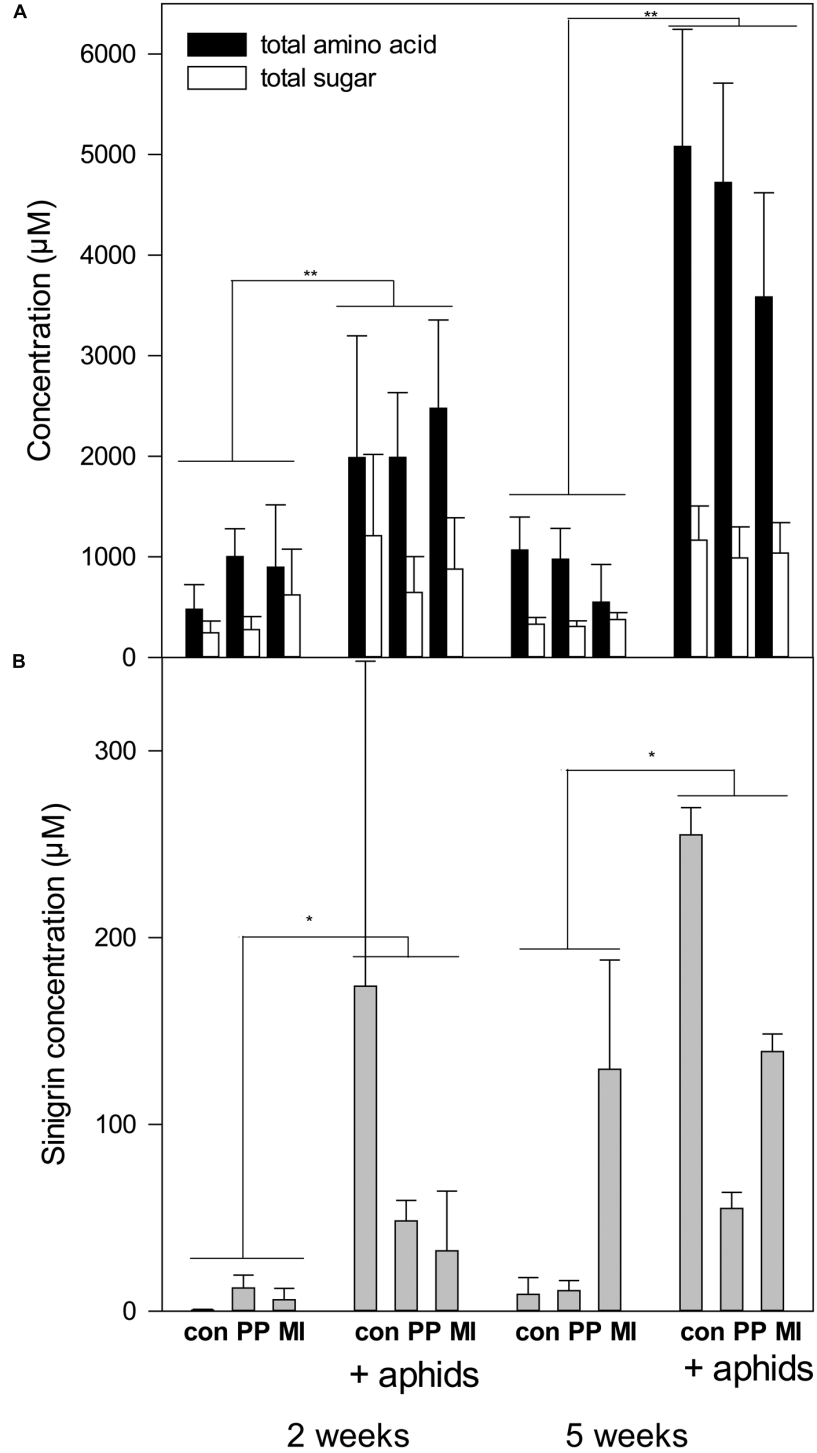
**Concentrations of amino acids, sugars and glucosinolates on plants with nematodes and aphids after 2 and 5 weeks. con, control; PP, *P. penetrans*; MI, *M. incognita*, aphids*, Brevicoryne brassicae*. (A)** Average (+SE) concentration of amino acids and sugars in phoem (*n* = 7 for 2 weeks, *n* = 8–11 for 5 weeks). Asterisks indicate a significant (^∗∗^*P* < 0.001, ^∗^*P* < 0.05) aphid treatment effect. For complete statistical results see **Table 4. (B)** Average (+SE) concentration of sinigrin in pooled phloem samples (*n* = 2–3). For statistical results see Supplementary Table S2.

In the experiments, the concentrations of amino acids and sugars in the phloem were strongly increased in the presence of aphids (**Figure 2A**, **Table 4**). This started already at the first harvest (after 2 weeks), with trends for sugars, and became very clear at the final harvest (after 5 weeks). Also the glucosinolate sinigrin was induced by the presence of aphids (*F*_1,17_ = 6.47 *P* = 0.02, **Figure 2B**, Supplementary Table S2). Pooling of the samples probably did not affect the outcome except for a decrease in statistical power, since the results were comparable between pooled and unpooled data for the amino acids and sugars (compare **Figure 2A** and Supplementary Figure S4). Nematodes did not significantly impact any of the measured chemical parameters and also the two nematode species did not differ in their effects on chemical parameters. The concentrations of amino acids, sugars and glucosinolates in the leaves did not differ significantly between treatments (Supplementary Table S3).

**Table 4:** MANOVA table showing the results from the analysis of the chemical composition of the phloem for amino acids and soluble sugar concentrations.

	Df	SS	MS	*F*	*P*
**Amino acids**					
Intercept	1	13.64	13.64	154.42	0
Nematodes	2	0.10	0.05	0.59	0.55
Aphids	1	1.93	1.93	21.87	<0.001
Harvest	1	0.49	0.49	5.51	0.02
Nema^∗^Aphids	2	0.05	0.02	0.26	0.77
Nema^∗^Harvest	2	0.21	0.11	1.21	0.30
Aphids^∗^Harvest	1	0.30	0.30	3.37	0.07
Nema^∗^Aphids^∗^Harvest	2	0.06	0.03	0.34	0.71
Error	79	6.98	0.09		
Total	90	10.68			
**Soluble sugars**					
Intercept	1	4.04	4.04	167.92	0
Nematodes	2	0.02	0.01	0.49	0.61
Aphids	1	0.56	0.56	23.23	<0.001
Harvest	1	<0.01	<0.01	0.15	0.70
Nema^∗^Aphids	2	0.03	0.01	0.55	0.58
Nema^∗^Harvest	2	0.05	0.03	1.06	0.35
Aphids^∗^Harvest	1	0.04	0.04	1.47	0.23
Nema^∗^Aphids^∗^Harvest	2	0.04	0.02	0.84	0.44
Error	79	1.90	0.02		
**Total**	90	2.72			

*n = 7 at harvest 2 weeks and n = 8–11 at harvest 5 weeks.*

**FIGURE 3:**
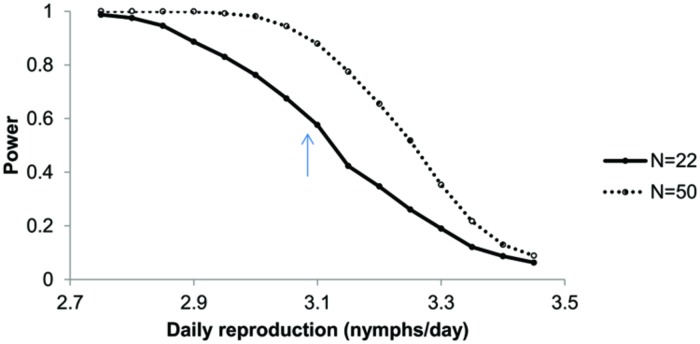
**Power analysis as a function of daily reproduction of *Brevicoryne brassicae* on plants with *Pratylenchus* in comparison to a Control treatment without nematodes, with a daily reproduction of 3.50 nymphs per day.** Daily reproduction on plants with *Pratylenchus* is varied from 2.75 to 3.45. Shown is the proportion of significant ANOVAs (*P* < 0.05) out of 1000 simulations for *n* = 22 and *n* = 50 per treatment. The arrow indicates the expected statistical power when daily reproduction of aphids on plants with *Pratylenchus* equals those observed in the experiment (3.08 nymphs/day).

The experimentally assessed life-history parameters were used in an individual-based simulation model for aphid population growth. The model revealed that the differences in reproduction rate per female were sufficient to result in a similar pattern as was found in the experiments: with all other parameters equal, modeled aphid populations developed the slowest on plants infested with *P. penetrans* compared to the control and *Meloidogyne* plants (**Figure 1B**). After 10 days the numbers of aphids counted in the experiment and obtained in the simulation model were similar in magnitude (approximately 50 aphids per plant). After this time point, the numbers of aphids in the simulation model increased much faster than in the greenhouse experiment (**Figure 1B**). A power analysis of the simulation models showed that it is hard to detect a significant difference in aphid population numbers after 14 days, assuming a difference between control and nematode treatments in daily reproduction of 0.42 nymphs per day and using 22 replicates (**Figure 3**).

## Discussion

Our results showed that the effect of nematodes on *B. brassicae* population depends on the nematode species; aphids populations developed more slowly on plants infested with the migratory endoparasitic nematode *P. penetrans*, but were not affected by the inoculation with the sedentary endoparasitic nematode *M. incognita*. Negative effects of *Pratylenchus* spp. on aphid numbers have been found before (Wurst and van der Putten, 2007), but the same applies to *Meloidogyne* spp. (Sell and Kuo-Sell, 1990) and other sedentary endoparasitic nematodes (Kaplan et al., 2011; Hol et al., 2013) on a range of plant species and various aphids. Here we explicitly compared the effects of two nematodes on a single wild plant species infested with one of its natural herbivores. Thus in this case the difference in feeding strategy of the two nematodes species is more likely the explanation for their different effects on *B. brassicae*.

The lower number of aphids on *Pratylenchus*-infested plants could be caused by a decrease in fecundity, delayed maturation or increased mortality. In the life-history experiment the fecundity (nymphs⋅reproductive female^–1^⋅day^–1^) was the only parameter that differed between *Pratylenchus* and control plants. With the simulation model we could demonstrate that this small difference, 0.42 nymphs per female per day or a ∼13% decrease, in fecundity would lead to significant differences in population numbers in about 60% of the cases. To detect this small difference in fecundity in 95% of all cases, the number of replicates would have to be around 50. In line with Peck (2004) and Meyer et al. (2009a), this demonstrates how a combination of short-term life history experiments with a model could be useful for developing a powerful experimental design. In addition, comparison of the outcome of the model with the actual aphid population size can also indicate the likelihood of other parameters being important to explain the effects. In our case, the model suggests that changes in fecundity alone would lead to exponential growth, rather than the linear population development that was observed in the greenhouse experiment. The difference between the model output and the results from the greenhouse experiment could be due to density-dependent effects that occur in real aphid populations (Menendez et al., 2013), which are not incorporated in the model and may have affected life history parameters as well. The life-history experiment was performed with only two reproducing adults per plant, with daily removal of nymphs, and thus the plants were always exposed to a low density of aphids. In the aphid population experiment plants were infested with five individuals, which after 10 days already became 50 individuals. The rapid increase in numbers may have crossed a threshold causing induced defense, which might not have happened in the life history experiment due to the low densities. Additional experiments, with assessments of life-history parameters on already aphid-infected plants are needed to test this, potentially in combination with corresponding model scenarios.

Induced responses indeed may play a role in this experiment. Analysis of sinigrin levels in the phloem showed that aphids increased sinigrin concentrations. Nematodes alone showed no significant effects on sinigrin concentrations; they appeared to reduce the increase in sinigrin levels on plants with aphids but this was not supported by the statistical analysis. *B. brassicae* being a specialist on Brassicaceae is known to store glucosinolate in its body for its own defense (Hopkins et al., 2009). Consequently, for this species it may rather serve as a feeding stimulant than a feeding deterrent. Thus, if induced defense played a role, it must have been via other defense mechanisms than the glucosinolates, e.g., flavonoids, camalexin or callose depositions in sieve elements (Kuśnierczyk et al., 2008; Mewis et al., 2012). The effect of aphids on sinigrin concentrations in the phloem was unaffected by nematode presence and thus changes in induced defense are not a causal explanation for the differences between *Pratylenchus* and *Meloidogyne* on aphid numbers. Alternatively, nematodes species could have differentially altered source-sink relations in the plant. Aphids apparently created a strong sink, increasing sugars and amino acid level in the phloem, but this was again not affected by nematode presence. This is in line with Kaplan et al. (2011), who found that *M. incognita* did not affect *M. persicae* sinks. Here we could show that the same applies to a nematode species with a different feeding strategy, the migratory endoparasite *Pratylenchus* and to the specialist aphid *B. brassicae*. It remains an open question why those two nematode species, which did not differ in their impacts on the measured chemical parameters, still had such diverging effects on aphid numbers. Among the factors known to affect aphid fecundity are nitrogen (Zarghami et al., 2010; Stafford et al., 2012), vitamin C (Kerchev et al., 2013), and moisture (Tariq et al., 2012). Nematodes can reduce nitrogen concentration and moisture content in plants (Hol et al., 2013), yet to our knowledge there is no evidence yet for differences between *Pratylenchus* and *Meloidogyne* in those effects.

It is well-known that greenhouse experiments may differ from what happens in the field (Vandegehuchte et al., 2010). For our study system it is known that *Pratylenchus* spp. commonly occur on *B. nigra* plants in the field, and thus it is quite likely that *B. brassicae* will encounter plants which are already infested with nematodes. The observed negative effect of root-lesion nematode presence on aphid numbers might not occur if aphids are able to avoid nematode-infected plants, if possible (Soler et al., 2009). Even if the aphids do not discriminate between nematode-infested and uninfested plants, this does not guarantee that nematode infested plants in the field will have smaller aphid populations than uninfested plants. The presence of other aphid species or higher trophic levels could modify the negative effect of nematodes on aphids. Competition between *M. persicae* and *B. brassicae* does depend on host plant quality (Stacey and Fellowes, 2002) and thus may depend on nematode presence. Some predators prefer to target plants with the highest aphid densities (Chaplin-Kramer et al., 2011) and thus aphids on nematode-infested plants might suffer less predation.

## Conclusion

By combining an experimental and modeling approach we could show relatively small differences in reproductive output caused by two nematode species with different feeding strategies, are sufficient to explain differences in aphid population development. Both nematode species had similar effects on chemical plant quality and eliminate a straightforward mechanistic role of sinigrin, amino acids and sugars in mediating these effects.

## Author Contributions

NvD originally formulated the idea and designed the experiments. CR, IM performed the greenhouse experiments. CR performed the chemical analyses. NvD, WH did the statistical analyses. WH, KM developed the individual based model. WH, NvD, KM wrote the manuscript; IM, CR provided editorial advice.

## Conflict of Interest Statement

The authors declare that the research was conducted in the absence of any commercial or financial relationships that could be construed as a potential conflict of interest.

## References

[B1] Bezemer T. M. De Deyn G. B. Bossinga T. M. van Dam N. M. Harvey J. A. Van der Putten W. H. (2005). Soil community composition drives aboveground plant-herbivore-parasitoid interactions. *Ecol. Lett.* 8 652–661. 10.1111/j.1461-0248.2005.00762.x

[B2] Bidart-Bouzat M. G. Kliebenstein D. (2011). An ecological genomic approach challenging the paradigm of differential plant responses to specialist versus generalist insect herbivores. *Oecologia* 167 677–689. 10.1007/s00442-011-2015-z 21625984

[B3] Bischoff A. Hurault B. (2013). Scales and drivers of local adaptation in *Brassica nigra* (Brassicaceae) populations. *Am. J. Bot.* 100 1162–1170. 10.3732/ajb.1200500 23720429

[B4] Brinkman E. P. van Veen J. A. van der Putten W. H. (2004). Endoparasitic nematodes reduce multiplication of ectoparasitic nematodes, but do not prevent growth reduction of *Ammophila arenaria* (L.) Link (marram grass). *Appl. Soil Ecol.* 27 65–75. 10.1016/j.apsoil.2004.02.004

[B5] Bruinsma M. Lucas-Barbosa D. ten Broeke C. J. M. van Dam N. M. van Beek T. A. Dicke M. (2014). Folivory affects composition of nectar, floral odor and modifies pollinator behavior. *J. Chem. Ecol.* 40 39–49. 10.1007/s10886-013-0369-x 24317664

[B6] Chaplin-Kramer R. Kliebenstein D. J. Chiem A. Morrill E. Mills N. J. Kremen C. (2011). Chemically mediated tritrophic interactions: opposing effects of glucosinolates on a specialist herbivore and its predators. *J. Appl. Ecol.* 48 880–887. 10.1111/j.1365-2664.2011.01990.x

[B7] De Deyn G. B. Raaijmakers C. E. Zoomer H. R. Berg M. P. de Ruiter P. C. Verhoef H. A. (2003). Soil invertebrate fauna enhances grassland succession and diversity. *Nature* 422 711–713. 10.1038/nature01548 12700759

[B8] de Vos M. Jander G. (2009). *Myzus persicae* (green peach aphid) salivary components induce defence responses in *Arabidopsis thaliana*. *Plant Cell Environ.* 32 1548–1560. 10.1111/j.1365-3040.2009.02019.x 19558622

[B9] Ellis P. R. Singh R. Pink D. A. C. Lynn J. R. Saw P. L. (1996). Resistance to *Brevicoryne brassicae* in horticultural brassicas. *Euphytica* 88 85–96. 10.1007/bf00032439

[B10] Erb M. Flors V. Karlen D. de Lange E. Planchamp C. D’Alessandro M. (2009). Signal signature of aboveground-induced resistance upon belowground herbivory in maize. *Plant J.* 59 292–302. 10.1111/j.1365-313X.2009.03868.x 19392694

[B11] Erb M. Meldau S. Howe G. A. (2012). Role of phytohormones in insect-specific plant reactions. *Trends Plant Sci.* 17 250–259. 10.1016/j.tplants.2012.01.003 22305233 PMC3346861

[B12] Erb M. Robert C. A. M. Hibbard B. E. Turlings T. C. J. (2011). Sequence of arrival determines plant-mediated interactions between herbivores. *J. Ecol.* 99 7–15. 10.1111/j.1365-2745.2010.01757.x

[B13] Gheysen G. Mitchum M. G. (2011). How nematodes manipulate plant development pathways for infection. *Curr. Opin. Plant Biol.* 14 415–421. 10.1016/j.pbi.2011.03.012 21458361

[B14] Grimm V. Berger U. Bastiansen F. Eliassen S. Ginot V. Giske J. (2006). A standard protocol for describing individual-based and agent-based models. *Ecol. Model.* 198 115–126. 10.1016/j.ecolmodel.2006.04.023

[B15] Grimm V. Berger U. DeAngelis D. L. Polhill J. G. Giske J. Railsback S. F. (2010). The ODD protocol: a review and first update. *Ecol. Model.* 221 2760–2768. 10.1016/j.ecolmodel.2010.08.019

[B16] Hol W. H. G. de Boer W. Termorshuizen A. J. Meyer K. M. Schneider J. H. van Dam N. M. (2010). Reduction of rare soil microbes modifies plant-herbivore interactions. *Ecol. Lett.* 13 292–301. 10.1111/j.1461-0248.2009.01424.x 20070364

[B17] Hol W. H. G. de Boer W. Termorshuizen A. J. Meyer K. M. Schneider J. H. Van Der Putten W. H. (2013). *Heterodera schachtii* nematodes interfere with aphid-plant relations on *Brassica oleracea*. *J. Chem. Ecol.* 39 1193–1203. 10.1007/s10886-013-0338-4 24014097 PMC3790247

[B18] Hol W. H. G. Raaijmakers C. E. Mons I. Meyer K. M. van Dam N. M. (2016). Data from: root-lesion nematodes suppress cabbage aphid population development by reducing aphid daily reproduction. (in press). 10.5061/dryad.s8r4n PMC4748742 26904074

[B19] Holm S. (1979). A simple sequentually rejective multiple test procedure. *Scand. J. Stat.* 6 65–70. 10.1016/j.jconhyd.2011.10.004

[B20] Hopkins R. J. van Dam N. M. van Loon J. J. A. (2009). Role of glucosinolates in insect-plant relationships and multitrophic interactions. *Ann. Rev. Entomol.* 54 57–83. 10.1146/annurev.ento.54.110807.090623 18811249

[B21] Hughes R. D. (1963). Population dynamics of the cabbage aphid, *Brevicoryne brassicae* (L.). *J. Anim. Ecol.* 32 393–424. 10.2307/2600

[B22] Johnson S. N. Clark K. E. Hartley S. E. Jones T. H. McKenzie S. W. Koricheva J. (2012). Aboveground-belowground herbivore interactions: a meta-analysis. *Ecology* 93 2208–2215. 10.1890/11-2272.1 23185882

[B23] Jones J. T. Haegeman A. Danchin E. G. Gaur H. S. Helder J. Jones M. G. (2013). Top 10 plant-parasitic nematodes in molecular plant pathology. *Mol. Plant Pathol.* 14 946–961. 10.1111/mpp.12057 23809086 PMC6638764

[B24] Kaplan I. Halitschke R. Kessler A. Sardanelli S. Denno R. F. (2008). Constitutive and induced defenses to herbivory in above– and belowground plant tissues. *Ecology* 89 392–406. 10.1890/07-0471.1 18409429

[B25] Kaplan I. Sardanelli S. Rehill B. J. Denno R. F. (2011). Toward a mechanistic understanding of competition in vascular-feeding herbivores: an empirical test of the sink competition hypothesis. *Oecologia* 166 627–636. 10.1007/s00442-010-1885-9 21181415

[B26] Kerchev P. I. Karpińska B. Morris J. A. Hussain A. Verrall S. R. Hedley P. E. (2013). Vitamin C and the abscisic acid-insensitive 4 transcription factor are important determinants of aphid resistance in *Arabidopsis*. *Antioxid. Redox Signal.* 18 2091–2105. 10.1089/ars.2012.5097 23343093

[B27] Kuśnierczyk A. Winge P. Jørstad T. S. Troczyńska J. Rossiter J. T. Bones A. M. (2008). Towards a global understanding of plant defence against aphids – timing and dynamics of early *Arabidopsis* defence responses to cabbage aphid (*Brevicoryne brassicae*) attack. *Plant Cell Environ.* 31 1097–1115. 10.1111/j.1365-3040.2008.01823.x 18433442

[B28] Kutyniok M. Müller C. (2012). Crosstalk between above– and belowground herbivores is mediated by minute metabolic responses of the host *Arabidopsis thaliana*. *J. Exp. Bot.* 63 6199–6210. 10.1093/jxb/ers274 23045608 PMC3481212

[B29] Lankau R. A. (2007). Specialist and generalist herbivores exert opposing selection on a chemical defense. *New Phytol.* 175 176–184. 10.1111/j.1469-8137.2007.02090.x 17547677

[B30] Lashkari M. R. Sahragard A. Ghadamyari M. (2007). Sublethal effects of imidacloprid and pymetrozine on population growth parameters of cabbage aphid, *Brevicoryne brassicae* on rapeseed, *Brassica napus* L. *Insect Sci.* 14 207–212. 10.1111/j.1744-7917.2007.00145.x

[B31] Le Guigo P. Rolier A. Le Corff J. (2012). Plant neighborhood influences colonization of Brassicaceae by specialist and generalist aphids. *Oecologia* 169 753–761. 10.1007/s00442-011-2241-4 22218942

[B32] Maffei M. E. Arimura G. I. Mithofer A. (2012). Natural elicitors, effectors and modulators of plant responses. *Nat. Prod. Rep.* 29 1288–1303. 10.1039/c2np20053h 22918379

[B33] Meldau S. Erb M. Baldwin I. T. (2012). Defence on demand: mechanisms behind optimal defence patterns. *Ann. Bot.* 110 1503–1514. 10.1093/aob/mcs212 23022676 PMC3503495

[B34] Menendez A. I. Folcia A. M. Vizgarra L. Romero A. M. Martinez-Ghersa M. A. (2013). Impact of plant and aphid stress history on infestation in arugula plants. *Entomol. Exp. Appl.* 149 128–137. 10.1111/eea.12116

[B35] Mewis I. Schreiner M. Nguyen C. N. Krumbein A. Ulrichs C. Lohse M. (2012). UV-B irradiation changes specifically the secondary metabolite profile in *Broccoli sprouts*: induced signaling overlaps with defense response to biotic stressors. *Plant Cell Physiol.* 53 1546–1560. 10.1093/pcp/pcs096 22773681 PMC3439869

[B36] Meyer K. M. Mooij W. M. Vos M. Hol W. H. G. van der Putten W. H. (2009a). The power of simulating experiments. *Ecol. Model.* 220 2594–2597. 10.1016/j.ecolmodel.2009.06.001

[B37] Meyer K. M. Vos M. Mooij W. M. Hol W. H. G. Termorshuizen A. J. Vet L. E. M. (2009b). Quantifying the impact of above- and belowground higher trophic levels on plant and herbivore performance by modeling. *Oikos* 118 981–990. 10.1111/j.1600-0706.2009.17220.x

[B38] Pavela R. Barnet M. Kocourek F. (2004). Effect of azadirachtin applied systemically through roots of plants on the mortality, development and fecundity of the cabbage aphid (*Brevicoryne brassicae*). *Phytoparasitica* 32 286–294. 10.1007/bf02979823

[B39] Peck S. L. (2004). Simulation as experiment: a philosophical reassessment for biological modeling. *Trends Ecol. Evol.* 19 530–534. 10.1016/j.tree.2004.07.019 16701318

[B40] Pieterse C. M. J. Van der Does D. Zamioudis C. Leon-Reyes A. Van Wees S. C. M. (2012). Hormonal modulation of plant immunity. *Annu. Rev. Cell Dev. Biol.* 28 489–521. 10.1146/annurev-cellbio-092910-154055 22559264

[B41] R Core Development Team (2013). *R: A Language and Environment for Statistical Computing* 3.0.0 Edn. Vienna: R Foundation for Statistical Computing.

[B42] Sell P. Kuo-Sell H.-L. (1990). Influence of infestation of oats by root nematodes (*Meloidogyne* sp.) on the performance of the cereal aphid, *Metopolophium dirhodum* (Walk.) (Hom., Aphididae). *J. Appl. Entomol.* 109 37–43. 10.1111/j.1439-0418.1990.tb00016.x

[B43] Soler J. J. Schaper S. V. Bezemer T. M. Cortesero A. M. Hoffmeister T. S. van der Putten W. H. (2009). Influence of presence and spatial arrangement of belowground insects on host-plant selection of aboveground insects: a field study. *Ecol. Entomol.* 34 339–345. 10.1111/j.1365-2311.2008.01082.x

[B44] Stacey D. A. Fellowes M. D. E. (2002). Influence of elevated CO2 on interspecific interactions at higher trophic levels. *Glob. Change Biol.* 8 668–678. 10.1046/j.1365-2486.2002.00506.x

[B45] Stafford D. B. Tariq M. Wright D. J. Rossiter J. T. Kazana E. Leather S. R. (2012). Opposing effects of organic and conventional fertilizers on the performance of a generalist and a specialist aphid species. *Agric. For. Entomol.* 14 270–275. 10.1111/j.1461-9563.2011.00565.x

[B46] Tariq M. Rossiter J. T. Wright D. J. Staley J. T. (2013). Drought alters interactions between root and foliar herbivores. *Oecologia* 172 1095–1104. 10.1007/s00442-012-2572-9 23292454

[B47] Tariq M. Wright D. J. Rossiter J. T. Staley J. T. (2012). Aphids in a changing world: testing the plant stress, plant vigour and pulsed stress hypotheses. *Agric. For. Entomol.* 14 177–185. 10.1111/j.1461-9563.2011.00557.x

[B48] van Bezooijen J. (2006). *Methods and Techniques for Nematology.* Wageningen: Wageningen University and Research Centre, 21–23.

[B49] van Dam N. M. Heil M. (2011). Multitrophic interactions below and above ground: en route to the next level. *J. Ecol.* 99 77–88. 10.1111/j.1365-2745.2010.01761.x

[B50] van Dam N. M. Oomen M. W. (2008). Root and shoot jasmonic acid applications differentially affect leaf chemistry and herbivore growth. *Plant Signal. Behav.* 3 91–98. 10.4161/psb.3.2.5220 19516980 PMC2633990

[B51] van Dam N. M. Raaijmakers C. E. van der Putten W. H. (2005). Root herbivory reduces growth and survival of the shoot feeding specialist *Pieris rapae* on *Brassica nigra*. *Entomol. Exp. Appl.* 115 161–170. 10.1111/j.1570-7458.2005.00241.x

[B52] Vandegehuchte M. L. De La Peña E. Bonte D. (2010). Interactions between root and shoot herbivores of *Ammophila arenaria* in the laboratory do not translate into correlated abundances in the field. *Oikos* 119 1011–1019. 10.1111/j.1600-0706.2009.18360.x

[B53] Will T. Furch A. C. U. Zimmermann M. R. (2013). How phloem-feeding insects face the challenge of phloem-located defenses. *Front. Plant Sci.* 4:336 10.3389/fpls.2013.00336 PMC3756233 24009620

[B54] Wondafrash M. Van Dam N. M. Tytgat T. O. G. (2013). Plant systemic induced responses mediate interactions between root parasitic nematodes and aboveground herbivorous insects. *Front. Plant Sci.* 4:87 10.3389/fpls.2013.00087 PMC3624084 23630532

[B55] Wurst S. van der Putten W. H. (2007). Root herbivore identity matters in plant-mediated interactions between root and shoot herbivores. *Basic Appl. Ecol.* 8 491–499. 10.1016/j.baae.2006.09.015

[B56] Zarghami S. Allahyari H. Bagheri M. R. Saboori A. (2010). Effect of nitrogen fertilization on life table parameters and population growth of *Brevicoryne brassicae*. *Bull. Insectol.* 63 39–43.

