# Supplementary material for: Root-Lesion Nematodes Suppress Cabbage Aphid Population Development by Reducing Aphid Daily Reproduction
Source: Front Plant Sci. 2016 Feb 10;7:111. doi: 10.3389/fpls.2016.00111 (PMC4748742; doi:10.3389/fpls.2016.00111)
Supplement: Supplementary file 1 [file Data_Sheet_1.DOCX]

**Supplementary Material for:**

**Root-lesion nematodes suppress cabbage aphid population development by reducing aphid daily reproduction**

W.H. Gera Hol, Ciska E. Raaijmakers, Ilse Mons, Katrin M. Meyer, Nicole M. van Dam

Email: [g.hol@nioo.knaw.nl](mailto:g.hol@nioo.knaw.nl)

**Methods**

Details of parameterization of the simulation model p 2

**Results**

Table S1 p 4

Table S2 p 5

Table S3 p 6

Fig. S1 p 7

Fig. S2 p 8

Fig. S3 p 9

Fig. S4 p 10

**Supplementary Material (separate): code for the simulation model**

**Methods**

For the three treatments (*Control, Meloidogyne, Pratylenchus*) in the life history experiment the probability of reproduction of a female was calculated in relation to age. The pattern is very similar for all treatments, between day 8 and 10 nearly all females will start to reproduce and the daily probability to reproduce stays continuously high (~95%) until the end of the experiment, day 16 (Online Resource 1 Fig. S1). In the simulation model we used the same reproduction probability for all treatments (*Control, Meloidogyne, Pratylenchus*) based on the similarities between treatments (Online Resource 1 Fig. S1). The data on daily reproduction from the life history experiment was used to fit several distributions and estimate parameters for the simulation model. For each plant (n=22) per treatment (Control, *Melodoigyne*, *Pratylenchus*) the sum of offspring of the two adults was expressed per day, ignoring the first day that nymphs were observed. Between day 8 and 16 in total 372 datapoints were collected for daily offspring production/2 adults. After Wilcoxon rank sum tests showed that the datasets differed between treatments (Control and *Pratylenchus* P = 0.024, *Meloidogyne* and *Pratylenchus* P = 0.014, Control and *Meloidogyne* P = 0.93), we fitted Poisson and Negative binomial distributions for the datasets separately per treatment, using the MASS package in R. For the Negative Binomial the overdispersion parameter size was estimated to be larger than ten and that means that the negative binomial is hard to differentiate from a Poisson distribution (compare lambda and mu in Online Resource 1 Figure S2 and S3). We choose to proceed with the Poisson distribution in the simulation model since it requires only one parameter to be estimated. For Control and *Meloidogyne* this means that on average 3.5 nymphs are produced per female per day, for *Pratylenchus* 3.08 and those values are used in the simulation model.

The simulation model was used to do a power analysis and test the frequency of significant differences between Control (no Nematodes) and *Pratylenchus* treatment. For Control treatments a daily reproduction of 3.5 nymphs per day was used and for the *Pratylenchus* we simulated a range from 2.75-3.45 (step 0.05) nymphs per day. One 1000 simulations were done for each data point in the range, producing 22 or 50 replicates per simulation for each treatment. The differences between the simulated datasets were tested with ANOVA in R. This shows that the power to detect a difference in daily reproduction of 0.45 nymphs per day is still rather limited for 22 replicates (Fig. 3 in the main text). For reasonable power (>95% significant tests) at least 50 replicates would be needed.

Supplementary Results - 3 Tables and 4 Figures

Table S1 Parametrization of the simulation model. Probability of aphids to reproduce depend on age, while daily reproduction depend on both age and the presence of nematodes

| Age (days) | Probability  to reproduce | Daily reproduction (aphid individuals) |
| --- | --- | --- |
| <8 | 0 | 0 |
| 8 | 0.02 | 2 |
| 9 | 0.2 | Max mean  Control 3.50, *Meloidogyne* 3.51, *Pratylenchus* 3.08 |
| 10 | 0.7 | Max mean |
| 11-18 | 0.95 | Max mean |
| 19-21 | 0.95 | Max mean-1 |
| >21 | 0 | 0 |

Table S2. ANOVA table showing the results from the analysis of sinigrin concentration in the phloem based on pooled samples. Samples were pooled per 3-4 samples, resulting in 2-3 datapoints per treatment.

|  | Df | SS | MS | F | P |
| --- | --- | --- | --- | --- | --- |
| Intercept | 1 | 1.25 | 1.25 | 39.85 | 0.00 |
| Nematodes | 2 | 0.03 | 0.01 | 0.45 | 0.65 |
| Harvest | 1 | 0.09 | 0.09 | 2.98 | 0.10 |
| Aphids | 1 | 0.20 | 0.20 | 6.47 | 0.02 |
| Nema*Aphids | 2 | 0.07 | 0.03 | 1.08 | 0.36 |
| Nema*Harvest | 2 | 0.13 | 0.07 | 2.10 | 0.15 |
| Aphids*Harvest | 1 | 0.00 | 0.00 | 0.03 | 0.86 |
| Nema*Aphids*Harvest | 2 | 0.01 | 0.01 | 0.21 | 0.82 |
| Error | 17 | 0.53 | 0.03 |  | |

Table S3. Concentrations (µmoles per gram dry mass) of soluble sugars (Sol. Sug.), amino acids (AA) and glucosinolates (GLS) in *Brassica nigra* leaves sampled 2 and 5 weeks after infestation with *Brevicoryne brassicae*. Plants were either inoculated with *Pratylenchus penetrans* or *Meloidogyne incognita* nematodes or mock inoculated (control) three weeks before the aphids were placed on the plant. The number of independent biological replicates is given as n in the table. Averages (+SE) are given.

| **Harvest after 2 weeks** | |  |  |  | |  | |  |
| --- | --- | --- | --- | --- | --- | --- | --- | --- |
| Treatment | Aphids? | n | Total Sol. Sug. | | Total AA | | Total GLS | |
| Control | NO | 7 | 77.3 (18.3) | | 264.9 (27.2) | | 26.0 (2.9) | |
| Pratylenchus | NO | 7 | 45.0 (9.3) | | 192.2 (31.4) | | 27.3 (3.7) | |
| Meloidogyne | NO | 6 | 74.9 (15.8) | | 218.6 (30.5) | | 23.6 (3.9) | |
|  |  |  |  | |  | |  | |
| Control | YES | 7 | 66.8 (16.5) | | 221.7 (29.6) | | 25.0 (2.0) | |
| Pratylenchus | YES | 7 | 76.0 (15.2) | | 248.9 (33.1) | | 25.3 3.6) | |
| Meloidogyne | YES | 7 | 67.5 (13.2) | | 182.5 (34.2) | | 26.7 (5.0) | |
|  |  |  |  | |  | |  | |
| **Harvest after 5 weeks** | |  |  | |  | |  | |
| Treatment | Aphids? | n | Total Sol. Sug. | | Total AA | | Total GLS | |
| Control | NO | 10 | 88.0 (15.4) | | 342.2 (45.8) | | 10.9 (1.7) | |
| Pratylenchus | NO | 9 | 103.7 (24.0) | | 286.1 (40.1) | | 7.0 (2.1) | |
| Meloidogyne | NO | 8 | 70.9 (10.4) | | 236.9 (21.8) | | 12.8 (4.0) | |
|  |  |  |  | |  | |  | |
| Control | YES | 9 | 131.3(23.5) | | 415.8 (45.0) | | 7.9 (2.0) | |
| Pratylenchus | YES | 11 | 83.7 (9.9) | | 211.5 (19.8) | | 6.4 (1.6) | |
| Meloidogyne | YES | 10 | 61.9 (11.5) | | 154.6 (20.9) | | 9.8 (1.9) | |

Figure S1. The observed probability of reproduction of a *Brevicoryne brassicae* individual in relation to age and nematode treatment. Control = no nematodes, Pratylenchus = *Pratylenchus penetrans*, Meloidogyne = *Meloidogyne hapla*. Calculated as (number of plants with reproducing females/total number of plants with females). Plants contained two clip cages, and the average values per plant were used. Absent individuals (either missing or dead) individuals were excluded from the dataset starting the day of absence; as a consequence the total number of independent replicates per treatment declined from 22-23 for day 1 to 17-20 for day 16.


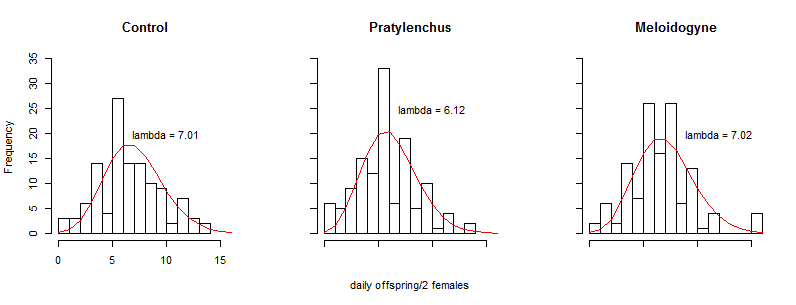


Figure S2. The observed daily reproduction of two reproducing females on one plant, with fitted Poisson distributions for the treatments without nematodes (Control), with *Pratylenchus penetrans* and with *Meloidogyne hapla.* The total number of daily reproduction events is 118 for Control and 127 for *Pra* and *Mel,* based on observations of females for multiple days on 22 plants per treatment.


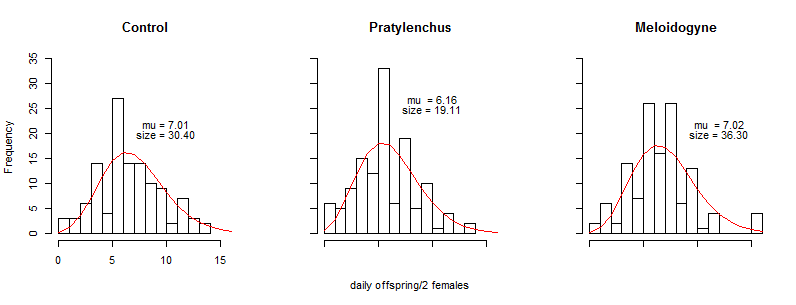


Figure S3. The observed daily reproduction of two reproducing females on one plant, with fitted Negative Binomial distributions for the treatments without nematodes (Control), with *Pratylenchus penetrans* and with *Meloidogyne hapla.* Number of datapoints is the same as in Fig S2.

Figure S4. Phloem concentrations of sugars and amino acids based on theoretically pooled samples, for comparison with Fig. 3b in the main text. Samples were pooled per 3-4 samples, resulting in 2-3 datapoints per treatment. Con = no nematodes added, PP = *Pratylenchus penetrans*, MI =*Meloidogyne incognita*, Aphids = *Brevicoryne brassicae*
