# Supplementary material for: Root-Lesion Nematodes Suppress Cabbage Aphid Population Development by Reducing Aphid Daily Reproduction
Source: Front Plant Sci. 2016 Feb 10;7:111. doi: 10.3389/fpls.2016.00111 (PMC4748742; doi:10.3389/fpls.2016.00111)
Supplement: Supplementary file 2 [file Data_Sheet_2.DOCX]

**Supplementary Material for:**

**Linking effects of root nematodes on aphid life-history parameters with aphid population development**

W.H. Gera Hol, Ciska E. Raaijmakers, Ilse Mons, Katrin M. Meyer, Nicole M. van Dam

Email: [g.hol@nioo.knaw.nl](mailto:g.hol@nioo.knaw.nl)

//Supplement–code for aphid population growth model

//The aphid population growth model is written in C# with Microsoft Visual C# 2010 Express

//required libraries

using System;

using System.Collections.Generic;

using System.Linq;

using System.Text;

using System.IO;

//application with definitions of model classes and functions and the execution routine

namespace ConsoleApplication1

{

//class Aphid with the properties age and whether alive or not

class Aphid

{

public int age;

public bool alive;

}

//class Program with definitions of model parameters and functions

class Program

{

//model parameters

public static int deathday = 25; //maximum aphid age in days

public static int start_number_aphids = 5;

public static int duration_experiment_days = 14;

public static int replicates = 22;

// function init to initialize the model parameters and functions

public static void init(List<Aphid> aphid_population)

{

for (int i = 0; i < start_number_aphids; i++)

{

Aphid eva = new Aphid();

eva.age = GetRandomNumber(10,25); //initial aphids get a random age between minimum 10 and maximum 24 days

eva.alive = true;

aphid_population.Add(eva);

}

}

//initializes a new instance of the Random class, using the system clock to provide a seed value

private static readonly Random getrandom = new Random();

//ensure that only one thread can access the random number generator at a time

private static readonly object syncLock = new object();

//function GetRandomNumber to obtain a uniformly distributed random integer number between a minimum and a maximum value to be specified

public static int GetRandomNumber(int min, int max)

{

lock (syncLock)

{ // synchronize

return getrandom.Next(min, max);

}

}

//function dies to find out whether an aphid (to be specified) dies or not, depending on its age

public static bool dies(Aphid l)

{

//aphids of age 1 to 24 have a probability of 0.04 to die

if (l.age > 0 && l.age < 25)

{

int temp = GetRandomNumber(1, deathday+1);

if (temp == 1)

{

return true;

}

else return false;

}

else

{

//aphids of age 25 always die

if (l.age > 24)

{

return true;

}

else return false;

}

}

//function GetPoisson to call function PoissonSmall

public static int GetPoisson(double lambda)

{

return PoissonSmall(lambda);

}

//function PoissonSmall to obtain a random value from the Poisson distribution (Knuth 1969, for λ<30)

private static int PoissonSmall (double lambda)

{

double p = 1.0, L = Math.Exp(-lambda);

int k = 0;

do

{

k++;

p *= (GetRandomNumber(1, 1000000) / 1000000.0);

}

while (p > L);

return k - 1;

}

//function reproduce to determine the age-dependent number of offspring of each aphid (Ellis et al. 1996)

public static void reproduce(List<Aphid> aphid_population, int age)

{

int offspring = 0;

//aphids younger than 8 days do not reproduce

if (age < 8)

{

offspring = 0;

}

else

{

//8-days-old aphids reproduce with a probability of 0.02

//number of offspring is drawn from a Poisson distribution

if (age == 8)

{

int temp = GetRandomNumber(1, 51);

if (temp == 1)

{

offspring = GetPoisson(2);

}

}

else

{

//9-days-old aphids reproduce with a probability of 0.2

//number of offspring is drawn from a Poisson distribution

if (age == 9)

{

int temp = GetRandomNumber(1, 6);

if (temp == 1)

{

offspring = GetPoisson(3.51);//the number of offspring is changed depending on nematode presence

}

}

else

{

//10-days-old aphids reproduce with a probability of 0.7

//number of offspring is drawn from a Poisson distribution

if (age == 10)

{

int temp = GetRandomNumber(1, 101);

if (temp < 71)

{

offspring = GetPoisson(3.51);

}

}

else

{

//11- to 18-days-old aphids reproduce with a probability of 0.95

//number of offspring is drawn from a Poisson distribution

if (age > 10 && age < 19)

{

int temp = GetRandomNumber(1, 101);

if (temp < 96)

{

offspring = GetPoisson(3.51);

}

}

else

{

//19- to 21-days-old aphids reproduce with a probability of 0.95

//number of offspring is drawn from a Poisson distribution but lower than aphids of 10-18 days

if (age > 18 && age < 22)

{

int temp = GetRandomNumber(1, 101);

if (temp < 96)

{

offspring = GetPoisson(2.51);

}

}

else

{

//22- to 25-days-old aphids do not reproduce

if (age > 21 && age < 26)

{

{

offspring = 0;

}

}

else offspring = 0;

}

}

}

}

}

}

//According to the determined number of offspring, new aphids are produced and added to the population of aphids

for (int i = 0; i < offspring; i++)

{

Aphid eva = new Aphid();

eva.age = 0;

eva.alive = true;

aphid_population.Add(eva);

}

}

//function Main to execute the program

static void Main(string[] args)

{

string lines = "";

// loop over all replicates

for (int h = 0; h < replicates; h++)

{

//initialization of the list of aphids

List<Aphid> population = new List<Aphid>();

init(population);

int pop_size = 0; //counter for the number of alive aphids

//loop over the days of the experiment

for (int day = 0; day < duration_experiment_days; day++)

{

// loop over all aphids currently in the population

for (int aphidnumber = 0; aphidnumber < population.Count(); aphidnumber++)

{

//only alive aphids are considered

if (population[aphidnumber].alive == true)

{

//mortality: does this aphid die?

if (dies(population[aphidnumber]))

{

population[aphidnumber].alive = false;

}

//reproduction of this aphid

reproduce(population, population[aphidnumber].age);

//aging of this aphid by 1 day

population[aphidnumber].age++;

}

}

//counting the number of alive aphids

pop_size = 0;

for (int aphidnumber = 0; aphidnumber < population.Count(); aphidnumber++)

{

if (population[aphidnumber].alive == true)

{

pop_size++;

}

}

//Writing output

}

System.Console.WriteLine("aphid_population size end experiment " + h + " = " + pop_size.ToString());

lines += h + "," + pop_size.ToString() + "\r\n";

}

// Write the string to a file.

System.IO.StreamWriter file = new System.IO.StreamWriter("D:/Users/gerah/Desktop/Output_Aphids.csv");

file.WriteLine(lines);

file.Close();

System.Console.Read();

}

}

}
